# Potential of Gallium as an Antifungal Agent

**DOI:** 10.3389/fcimb.2019.00414

**Published:** 2019-12-11

**Authors:** Rafael Wesley Bastos, Luana Rossato, Clara Valero, Katrien Lagrou, Arnaldo Lopes Colombo, Gustavo H. Goldman

**Affiliations:** ^1^Faculdade de Ciências Farmacêuticas de Ribeirão Preto, Universidade de São Paulo, São Paulo, Brazil; ^2^Escola Paulista de Medicina, Universidade Federal de São Paulo, São Paulo, Brazil; ^3^Laboratory of Clinical Bacteriology and Mycology, Department of Microbiology and Immunology, KU Leuven, Leuven, Belgium

**Keywords:** *Aspergillus fumigatus*, *Candida*, gallium nitrate, multidrug resistant, iron

## Abstract

There are only few drugs available to treat fungal infections, and the lack of new antifungals, along with the emergence of drug-resistant strains, results in millions of deaths/year. An unconventional approach to fight microbial infection is to exploit nutritional vulnerabilities of microorganism metabolism. The metal gallium can disrupt iron metabolism in bacteria and cancer cells, but it has not been tested against fungal pathogens such as *Aspergillus* and *Candida*. Here, we investigate *in vitro* activity of gallium nitrate III [Ga(NO_3_)_3_] against these human pathogens, to reveal the gallium mechanism of action and understand the interaction between gallium and clinical antifungal drugs. Ga(NO_3_)_3_ presented a fungistatic effect against azole-sensitive and -resistant *A. fumigatus* strains (MIC_50/90_ = 32.0 mg/L) and also had a synergistic effect with caspofungin, but not with azoles and amphotericin B. Its antifungal activity seems to be reliant on iron-limiting conditions, as the presence of iron increases its MIC value and because we observed a synergistic interaction between gallium and iron chelators against *A. fumigatus*. We also show that an *A. fumigatus* mutant (Δ*hapX*) unable to grow in the absence of iron is more susceptible to gallium, reinforcing that gallium could act by disrupting iron homeostasis. Furthermore, we demonstrate that gallium has a fungistatic effect against different species of *Candida* ranging from 16.0 to 256.0 mg/L, including multidrug-resistant *Candida auris, C. haemulonii, C. duobushaemulonii*, and *C. glabrata*. Our findings indicate that gallium can inhibit fungal pathogens *in vitro* under iron-limiting conditions, showing that Ga(NO_3_)_3_ could be a potential therapy not only against bacteria but also as an antifungal drug.

## Introduction

Fungal infection is an underestimated threat that affects over one billion people with a mortality rate higher than 1.6 million of people per year, similar to that estimated for tuberculosis and several fold greater than malaria (Brown et al., 2012a; Almeida et al., 2019). The four most fatal fungi isolated during clinical practices are species of *Candida, Cryptococcus, Aspergillus*, and *Pneumocystis* (Brown et al., 2012b). These microorganisms can cause severe and often fatal systemic infections without appropriate therapy (Perfect, 2017).

Currently, there are few available drugs to treat severe fungal infections that belong to classes of azole, pyrimidine analogs, polyene, or echinocandin antifungals (Perfect, 2017). However, the disadvantages of current antifungals include high toxicity, limited capability to inhibit multiple fungal cell targets, rapid development of drug resistance when used in single therapy, and low half-life (Perfect and Bicanic, 2015; Nett and Andes, 2016; Perfect, 2017). These factors, combined with the emergence of new multidrug-resistant species such as *Candida auris*, may explain why therapies against severe mycoses remain unsatisfactory. In this context, it is essential to search and develop new antifungal drugs (Perfect, 2017).

Gallium is a group IIIA metal in the periodic table of elements with several medical applications including its use as a diagnostic and therapeutic agent in cancer and disorders of calcium and bone metabolism (Chitambar, 2010). Furthermore, it has been demonstrated that gallium compounds, such as Ga(NO3)3 (gallium nitrate III), have antibacterial effects against *Mycobaterium* (Olakanmi et al., 2000), *Francisella tulerens* (Olakanmi et al., 2009), *Acinetobacter baumannii* (Antunes et al., 2012), *Klebsiella pneumoniae* (Thompson et al., 2015), *Staphylococcus aureus* (Richter et al., 2017), and *Pseudomonas aeruginosa* (Goss et al., 2018).

Gallium has a nearly identical ionic radius to iron and can be taken up by cellular iron transport systems and can replace it in iron-containing proteins (Chitambar and Narasimhan, 1991; Goss et al., 2018). Unlike iron, gallium cannot be reduced in physiological conditions (Apseloff, 1999), which inhibits the functionality of gallium-complexed proteins and arrests cell growth (Goss et al., 2018). Thus, this element acts as a “Trojan horse” by disrupting the structure of proteins that incorporate iron in both bacteria and cancer cells (Chitambar, 2010; Goss et al., 2018).

Despite its known antibacterial and antitumoral activity, to the best of our knowledge, the antifungal effect of gallium has been poorly reported (Bastos et al., 2010). Here, we tested *in vitro* gallium activity against *A. fumigatus* and *Candida*, performed experiments aiming to understand gallium mechanism of action, and studied the interaction between gallium and systemic antifungal drugs.

## Materials and Methods

### Microorganisms, Media, and Drugs

All of the fungal strains used in this work are described in Table 1. Azole-resistant *Aspergillus fumigatus* strains were isolated from different sources in Belgium or Switzerland. Susceptible *Candida* strains are reference lineages or isolates from patients from São Paulo Hospital (Brazil) and belonging to the collection of the Special Mycology Laboratory, São Paulo Federal University. Multidrug-resistant *Candida* isolates were previously characterized by our group as resistant to at least two classes of antifungal drugs (Bizerra et al., 2014; Dal Mas et al., 2019). All strains were maintained in glycerol 10% at −80°C until they were used. Strains were grown either in YPD (1% w/v yeast extract and 2% peptone and dextrose) (yeast strains) or glucose minimal medium (GMM) (1% w/v glucose, 50 ml/L of a 20× salt solution, 1 ml/L of 5× trace elements, pH 6.5) (*A. fumigatus*) (Alves de Castro et al., 2016). Iron-depleted MM (adapted minimal medium, AMM) was prepared in the same manner as GMM except that all iron-containing compounds were taken out from the trace elements (Alves de Castro et al., 2016). If required, agar was added to a final concentration of 1.7% w/v or 2% w/v to the GMM or YPD, respectively. We also used RPMI-1640 test medium (Gibco) buffered with morpholinepropanesulfonic acid (Sigma-Aldrich). All growth was carried out at 37°C for 24–48 h.

**Table 1:** Fungal strains used.

**Strains**	**Site of isolation**	**Country**
*Aspergillus fumigatus* CEA10^(S)^	NA	NA
*A. fumigatus* CEA10 Δ*hapX*^[1]^	NA	NA
*A. fumigatus* Af293^(S)^	NA	NA
*A. fumigatus* AfS35^(S)^	NA	NA
*A. fumigatus* CEA17^(S)^	NA	NA
*A. fumigatus* CYP-15-75^(AR)^	Sputum	Belgium
*A. fumigatus* CYP-15-91^(AR)^	Sputum	Belgium
*A. fumigatus* CYP-15-93^(AR)^	Bronchoalveolar lavage	Belgium
*A. fumigatus* CYP-15-106^(AR)^	Sputum	Belgium
*A. fumigatus* CYP-15-108^(AR)^	Sputum	Belgium
*A. fumigatus* CYP-15-109^(AR)^	Sputum	Belgium
*A. fumigatus* CYP-15-115^(AR)^	Sputum	Belgium
*A. fumigatus* CYP-15-117^(AR)^	Sputum	Belgium
*A. fumigatus* CYP-15-146^(AR)^	Sputum	Belgium
*A. fumigatus* CYP-15-147^(AR)^	Bronchoalveolar lavage	Belgium
*A. fumigatus* 17993925^(AR)^^[2]^	Bronchoalveolar lavage	Switzerland
*A. fumigatus* 20089320^(AR)^^[2]^	Bronchoalveolar lavage	Switzerland
*Candida albicans* ATCC 90025^(S)^	NA	NA
*C. albicans* 16^(S)^	Blood culture	Brazil
*C. albicans* 83^(S)^	Blood culture	Brazil
*C. albicans* 106^(S)^	Blood culture	Brazil
*C. albicans* 123^(S)^	Blood culture	Brazil
*C. haemulonii sensu stricto* CBS 5149^(MDR)^^[3]^	Blood culture	NA
*C. haemulonii* sensu stricto 768^(S)^	Blood culture	Brazil
*C. haemulonii* sensu stricto 3834A^(S)^	Blood culture	Brazil
*C. haemulonii* sensu stricto 6083^(S)^	Blood culture	Brazil
*C. haemulonii* sensu stricto 145/18^(S)^	Blood culture	Brazil
*C. haemulonii* sensu stricto 585/2015(^MDR)^^[3]^	Blood culture	Brazil
*C. haemulonii* sensu stricto 767/2015^(MDR)^^[3]^	Catheter tip	Brazil
*C. haemulonii* sensu stricto 9700B^(MDR)^^[3]^	Blood culture	Brazil
*C. haemulonii* var *vulnera*^[^583/2015^(MDR)^^[3]^	Blood culture	Brazil
*C. haemulonii var vulnera* 9873 ^(MDR)^^[3]^	Blood culture	Brazil
*C. duobushaemulonii* 546/2015 ^(MDR)^^[3]^	Vaginal secretion	Brazil
*C. duobushaemulonii* 6983 ^(MDR)^^[3]^	Blood culture	Chile
*C. glabrata* ATCC 90030^(S)^	NA	NA
*C. glabrata* 614^(S)^	Blood culture	Brazil
*C. glabrata* 636^(S)^	Blood culture	Brazil
*C. glabrata* 558^(S)^	Blood culture	Brazil
*Candida glabrata* 8622 A^(MDR)^^[4]^	Blood culture	Brazil
*Candida glabrata* 8622 B^(MDR)^^[4]^	Blood culture	Brazil
*Candida glabrata* 8622 C^(MDR)^^[4]^	Blood culture	Brazil
*Candida glabrata* 8622 D^(MDR)^^[4]^	Blood culture	Brazil
*C. parapsilosis* ATCC 22019^(S)^	NA	NA
*C. krusei* ATCC 6558^(S)^	NA	NA
*C. auris* CBS 10913^(S)^	NA	NA
*C. auris* 470/2015^(MDR)^^[4]^	Blood culture	Brazil
*C. auris* 473/2015 ^(MDR)^^[4]^	Not informed	Venezuela
*C. auris* 484/2015^(MDR)^^[4]^	Blood culture	Brazil
*C. auris* 467/2015^(MDR)^^[4]^	Blood culture	Brazil
*C. auris* 490/2015 ^(MDR)^^[4]^	Blood culture	Venezuela
*C. auris* 501/2015 ^(MDR)^^[4]^	Urine	Venezuela
*C. auris* 502/2015 ^(MDR)^^[4]^	Urine	Venezuela

*S, susceptible; AR, azole-resistant; MDR, multidrug-resistant; NA, not applicable*.

*1, Alves de Castro et al. (2016); 2, Riat et al. (2018); 3, Dal Mas et al. (2019); 4, Bizerra et al. (2014)*.

We purchased gallium nitrate III [Ga(NO_3_)_3_] and systemic antifungal drugs, posaconazole, voriconazole, caspofungin, and amphotericin B from Sigma-Aldrich. Gallium nitrate and caspofungin were dissolved in water, and the other drugs were dissolved in dimethyl sulfoxide (DMSO) (Sigma-Aldrich).

### Antifungal Activity Assays

Minimum inhibitory concentration (MIC) of gallium nitrate was determined using RPMI-1640 based on M38-A2 (molds) (Clinical Laboratory Standards Institute, 2017a) or M27-A3 (Clinical Laboratory Standards Institute, 2017b) (yeasts) protocols of the Clinical and Laboratory Standards Institute (CLSI) broth microdilution for antifungal susceptibility assays. GMM and AMM also were used when stated. MIC was determined as the lowest concentration of gallium or other drugs that visually inhibited 100% (except when stated) fungal growth.

After performing the MIC assay, we checked if gallium nitrate has a fungistatic or fungicidal effect. Aliquots of 100 μl were removed from the wells where there was no visible growth and from the original inoculum and then subcultured in GMM or YPD and incubated at 37°C for 2 days. Samples were seeded in Petri dishes in duplicate. The compound was classified as fungicide if it was able to reduce 99% of fungal load comparing it with the initial inoculum; otherwise, it was considered fungistatic.

### Killing Assay

Killing curves were obtained by following a procedure described previously with some modifications (Öz et al., 2016). Briefly, microplates containing 1-, 4-, and 16-fold higher than the MIC of gallium, diluted in RPMI, were prepared. Then, 100 μl of an inoculum suspension at the concentration of 2 × 10^6^ was added to each well. The plates were incubated at 37°C for 2, 8, 16, 24, or 48 h before measurements of cellular viability with XTT-menadione. Each plate was taken from the incubator 2 h prior to the end of the incubation time, and 50 μl of XTT-menadione (1 mg/ml XTT with 125 μM menadione in saline) solution was added to each well. After 2 h of incubation with XTT-menadione, the plates were centrifuged (3,000 × *g*, 10 min), the supernatant was replaced in a new microplate, and the plates were read at 492 nm with a microplate reader. The experiment was performed with four replicates, and the result was evaluated by comparing the absorbance in the growth in the presence of gallium with the negative control conditions (without gallium).

### Antifungal Drug Combination Activity Assay

We tested combination between gallium nitrate and antifungal drugs against *A. fumigatus* conidia using a checkerboard microdilution method (Santos et al., 2006), which provides a matrix of all possible drug combinations in the required concentration range. The concentrations ranged from 0.125 to 64.0 mg/L for gallium, 0.125–8.0 mg/L for posaconazole and voriconazole, 4.0–256.0 mg/L for caspofungin, and 0.5–32.0 mg/L for amphotericin B. Briefly, 50 μl of each dilution of clinical antifungals and gallium was added to 96-well plates in the horizontal and vertical orientation, respectively. Then, 100 μl of the inoculum (1 × 10^4^ conidia/ml) was added to the plate containing various combinations of drug and gallium concentrations. The plates were incubated at 37°C during 48 h and one plate was used to test each strain. The MIC endpoint was 100% of growth inhibition. The interaction was quantitatively evaluated by determining the fractional inhibitory concentration index (FICI): FICI = [MIC gallium in combination/MIC gallium] + [MIC clinical drug in combination/MIC clinical drug]. The FICI was calculated for all of the possible combinations of different concentrations for the same isolate and the final result was expressed as the mean of the FICIs (Gomez-lopez et al., 2003). Also, interaction curves were constructed. The interaction between these drugs was classified as synergism if FICI ≤ 0.5, indifferent if 0.5 < FICI ≤ 4.0, and antagonism for FICI > 4.0 (Odds, 2003). Wells of the checkerboard plates with the combination between gallium and caspofungin were also recorded using Tucsen (ISH500) camera coupled to a Nikon (eclipse E100) microscope.

The interaction between iron and gallium was evaluated using the checkerboard methodology using RPMI-1640 and AMM media. First, the FICI for the interaction of gallium (0.125–64.0 mg/L) with the iron chelator Bathophenanthrolinedisulfonic acid (4,7-diphenyl-1,10-phenanthrolinedisulfonic acid [BPS] (Sigma-Aldrich) (7.8–500 μM) was calculated. Further, we combined different concentrations of gallium and FeSO_4_ (0.007–0.5% w/v) in microplates. After 48 h at 37°C, we analyzed the MIC of gallium in presence of iron and imaged the wells (plates with RPMI) as described before.

### Radial Growth and Caspofungin Paradoxical Effect (CPE) Test

To determine radial growth in the presence of gallium, wild-type and Δ*hapX* strains from *A. fumigatus* were grown from 10^5^ spores for 5 days on plates containing AMM supplemented with different concentrations of gallium (16.0–512.0 mg/L). Growth results were expressed as ratios, dividing colony radial diameter (cm) of growth in the gallium condition by colony radial diameter in the control (no gallium) (Ries et al., 2017).

In order to study if Ga(NO_3_)_3_ can interfere in CPE, we determined the radial growth of wild-type strain in AMM supplemented with caspofungin at a lower (1 mg/L) and higher (8 mg/L) concentration combined or not with gallium at sub-inhibitory concentration.

All radial growth experiment was done in triplicate.

### Statistical Analysis

All experiments were repeated at least twice. All statistical analyses were performed using GraphPad Prism, version 8.00, for Windows (GraphPad Software, San Diego, CA, USA) with *P* < 0.05 considered significant. CPE interference was analyzed by Student's *t*-test and radial growth in the presence of gallium by two-way ANOVA followed by Bonferroni test.

## Results

### Gallium Has a Fungistatic Effect Against Azole-Sensitive and -Resistant *Aspergillus fumigatus* Strains

We tested if gallium nitrate III [Ga(NO_3_)_3_] could inhibit *A. fumigatus* growth by determining the MIC in microdilution plates. We used azole-sensitive *A. fumigatus* strains common in laboratory practice (CEA10, CEA17, Af293, and AfS35) and also azole-resistant isolates, with different resistance mechanisms, cultured from different sample sites from patients from Belgium and Switzerland (Table 1) (MIC for azole drugs in Table S1). The most common azole-resistance mechanisms include amino acid substitutions in the target Cyp51A protein and tandem repeat sequence insertions at the *cyp51A* promoter (Hagiwara et al., 2016). The *cyp51A* gene is not mutated in the Belgian strains (except CYP-15-91 in which *cyp51A* was not sequenced), suggesting different mechanisms of azole resistance. In contrast, strains 1799392 and 20089320 isolated from Switzerland have TR_34_ tandem repeats at the *cyp51* promoter region and L98H amino acid replacement at the Cyp51A (Riat et al., 2018).

Initially, the antifungal effect of gallium was tested in two media, GMM and RPMI. Growth inhibition was observed only in RPMI, with both MIC_50_ and MIC_90_ = 32.0 mg/L (MIC values that inhibited 50 and 90% of the strains, respectively) (Table 2). Furthermore, azole-resistant strains had MIC one dilution higher than susceptible isolated when RPMI was used (Table 2). Subsequently, we plated the content of those wells where the fungi had not visibly grown after MIC assay in RPMI to determine whether gallium has a fungistatic or fungicidal effect. Ga(NO_3_)_3_ was classified as fungistatic as it did not reduce 99% of fungal load compared to initial inoculum for all of the strains.

**Table 2:** Minimum inhibitory concentration (MIC, mg/L) of gallium nitrate for *A. fumigatus* strains in glucose minimal medium (GMM), iron-depleted minimal medium (AMM), and RPMI.

**Strain/parameter**	**GMM**	**RPMI**	**AMM**
*Aspergillus fumigatus* CEA10^S^	>512.0	16.0	16.0
*A. fumigatus* Af293^S^	>512.0	16.0	32.0
*A. fumigatus* AfS35^S^	>512.0	16.0	16.0
*A. fumigatus* CEA17^S^	>512.0	16.0	32.0
*A. fumigatus* CYP-15-75^AR^	>512.0	16.0	32.0
*A. fumigatus* CYP-15-91A^AR^	>512.0	32.0	16.0
*A. fumigatus* CYP-15-93^AR^	>512.0	32.0	16.0
*A. fumigatus* CYP-15-106^AR^	>512.0	32.0	16.0
*A. fumigatus* CYP-15-108 ^AR^	>512.0	32.0	16.0
*A. fumigatus* CYP-15-109^AR^	>512.0	32.0	16.0
*A. fumigatus* CYP-15-115^AR^	>512.0	32.0	16.0
*A. fumigatus* CYP-15-117^AR^	>512.0	32.0	16.0
*A. fumigatus* 17993925	>512.0	32.0	32.0
*A. fumigatus* 20089320	>512.0	32.0	32.0
MIC_50_	>512.0	32.0	16.0
MIC_90_	>512.0	32.0	32.0

*S, susceptible; AR, azole-resistant; MIC_50/90_, values that inhibited 50 and 90% of the strains, respectively*.

### Kinetics of the Action of Gallium

**Figure 1:**
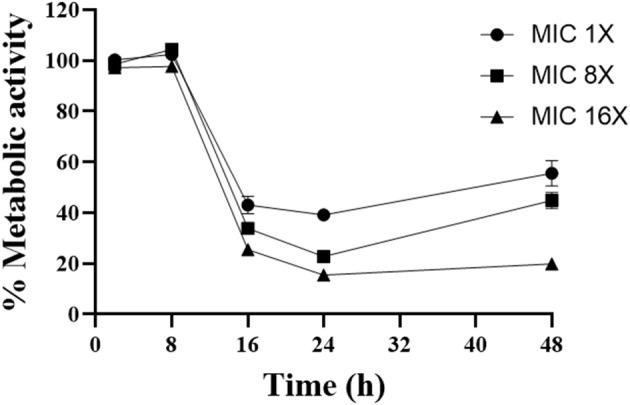
Time kill curve performed with different concentrations of gallium nitrate (1-, 4-, 16-fold MIC) against *A. fumigatus* CEA10.

To evaluate the kinetics of the action of gallium, *A. fumigatus* CEA10 was selected to test cell viability using XTT (Figure 1). We observed that increasing the gallium concentration has significant influence (*P* < 0.05) on the time to reduce the fungal viability. The best inhibitory effect of gallium was achieved at 24 h for all concentrations tested. At the highest concentration (MIC 16 ×), gallium was able to reduce 80% of the metabolic activity at 24 h, which was maintained until 48 h. However, at the other concentrations, part of metabolic activity was recovered after 24 h.

### Synergistic Effect Between Gallium and Caspofungin Against *A. fumigatus* CEA10

Combination of drugs is a potent alternative treatment used as a therapy against fungal infection (Perfect, 2017). In this context, drugs can have three types of interactions: antagonistic, indifferent, and synergistic (Odds, 2003). In order to study the effect of combining gallium with antifungal drugs used to treat aspergillosis (voriconazole, posaconazole, amphotericin B, and caspofungin), we determined the FICI.

**Figure 2:**
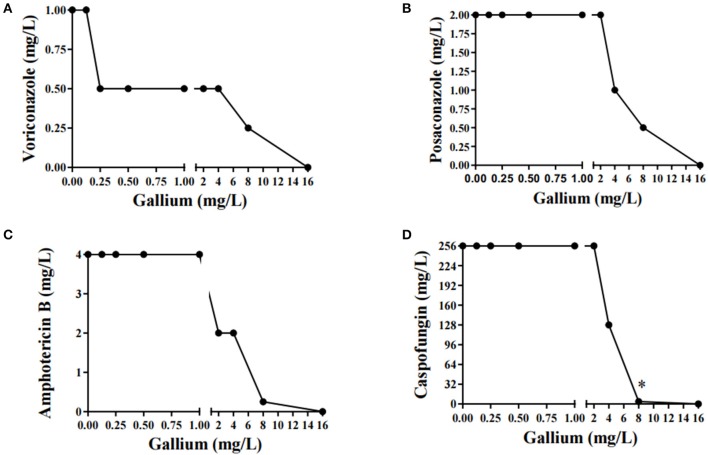
Combination curve of gallium and voriconazole **(A)**, posaconazole **(B)**, amphotericin B **(C)**, and caspofungin **(D)** against *A. fumigatus* CEA10. *synergism.

**Figure 3:**
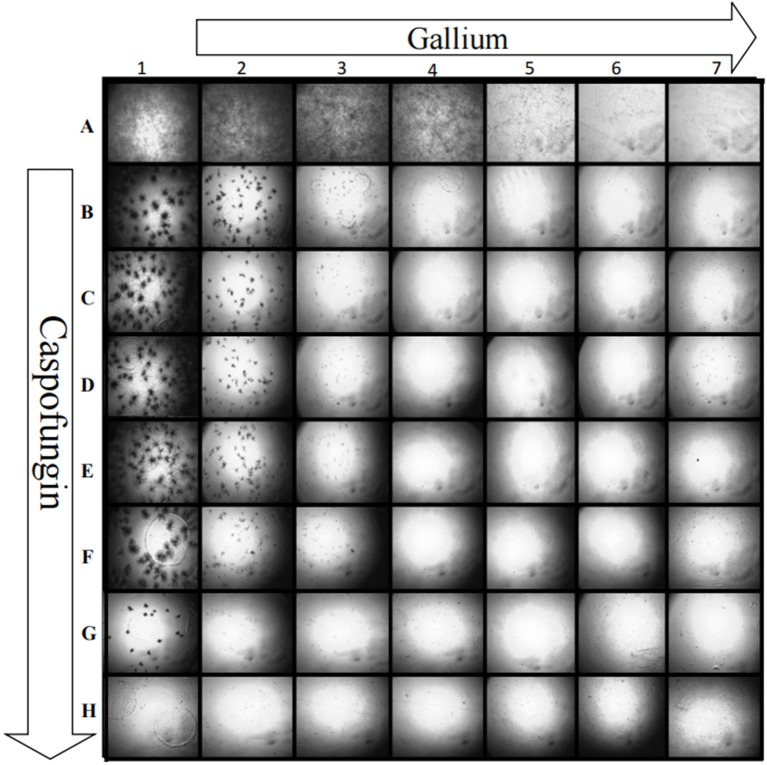
Growth of *A. fumigatus* CEA10 in checkerboard assay showing that when gallium (row) is combined with caspofungin (column), there is a visible decrease in the hyphae growth compared to the single drug challenge (column A and row 1). Image A1 shows the growth control, without drugs. Increase = 50×.

Gallium presented indifferent interactions with voriconazole, posaconazole, and amphotericin B (0.5 < FICI ≥ 4.0) (Figures 2A–C), but a synergistic interaction (FICI ≤ 0.5) with caspofungin (32.0 mg/L of gallium and 8 mg/L of caspofungin) (Figure 2D). This synergistic interaction became more evident when we imaged the wells from the checkerboard assay (Figure 3). When the conidia were challenged with both drugs (from column 2 and row B), hyphal growth decreased compared to the treatment with single drugs and the growth control (Figure 3).

### CPE Is Affected by Gallium

**Figure 4:**
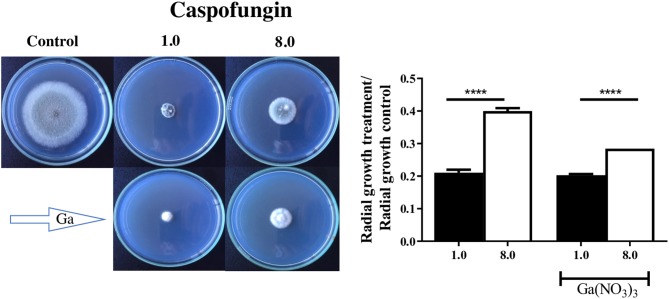
Gallium was combined with caspofungin at lower (1 mg/L) and higher (8 mg/L) concentrations. Radial growth indicates that gallium decreased caspofungin paradoxical effect in *A. fumigatus* CEA10. ****P < 0.0001.

Since gallium had a synergistic interaction with caspofungin in *A. fumigatus* CEA10, we evaluated whether it would affect the CPE, which is characterized by reduced activity of the drug at higher concentrations (Ries et al., 2017). In order to test that, first we determined the MIC of gallium in solid AMM. Then, we quantified the radial growth of the spores grown in AMM plus caspofungin, at lower (1 mg/L) and higher (8 mg/L) concentrations, and gallium at MIC/2 (64 mg/L) concentration. Figure 4 shows that *A. fumigatus* CEA10, independently of the presence of gallium, grew better at the highest concentration of caspofungin than at the lowest. However, the difference between the growth at lower and higher concentration of caspofungin is decreased by gallium (Figure 4), indicating that [Ga(NO_3_)_3_] interferes in CPE in *A. fumigatus* CEA10.

### Antifungal Effect of Gallium Is Dependent on Iron Concentration in the Medium

Our results have shown that gallium cannot inhibit *A. fumigatus* in GMM (MIC > 512.0 mg/L) (Table 2), suggesting that some components in the medium may interfere with gallium antifungal effect. In addition, it has been reported that gallium exerts its antibacterial and anticancer effect by disrupting iron metabolism (Chitambar, 2010; Goss et al., 2018). Thus, we investigated if the absence of inhibition in GMM would be related to the presence of iron in the medium by performing MIC tests in GMM without iron. In iron-depleted GMM, gallium inhibited *A. fumigatus* growth with MIC_50_ and MIC_90_ = 16.0 and 32.0 mg/L, respectively, similar values observed when the test was done in RPMI (Table 2).

**Figure 5:**
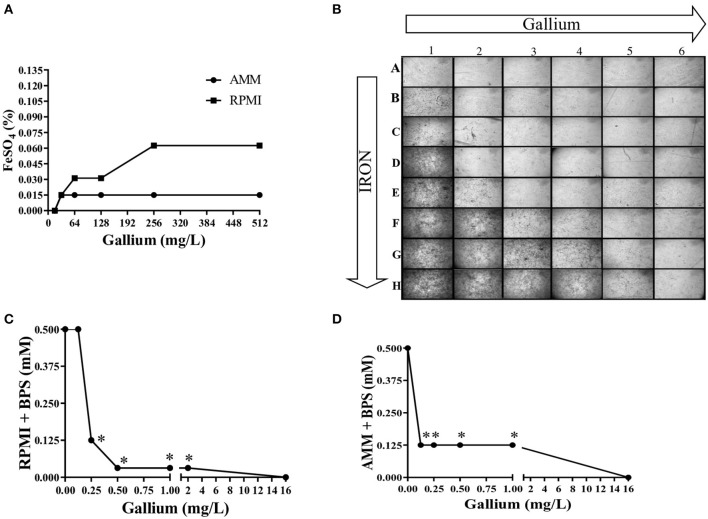
Iron presence interferes in gallium anti-*Aspergillus* effect. The MIC of gallium for against *A. fumigatus* CEA10 increases adding FeSO_4_ **(A,B)**. Combination between gallium and BPS (iron chelator) has a strong synergism in RPMI **(C)** and iron-depleted medium **(D)**. Increase = 50×. ^*^synergism.

To confirm that iron interferes with the anti-*Aspergillus* effect of gallium, conidia from *A. fumigatus* CEA10 were inoculated in microplates containing AMM and RPMI with combined concentrations of gallium and FeSO_4_. Figure 5A shows that the gallium concentration able to inhibit fungal growth in the absence of iron was 16.0 mg/L in both media. However, with iron addition, the gallium MIC increased, especially when RPMI was used (Figure 5A). Images from the wells (plates with RPMI) also confirmed that increased concentrations of iron negatively affect the antifungal activity of gallium against *A. fumigatus* CEA10 (Figure 5B).

### Combination Between Gallium and Iron-Chelator Is Synergistic in *A. fumigatus*

**Figure 6:**
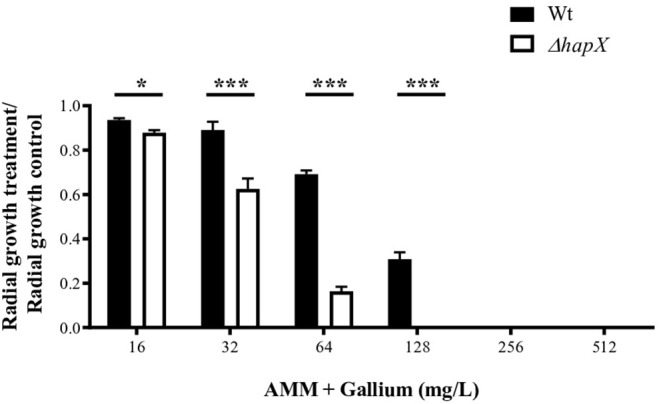
Radial growth of wt and Δ*hapX* in AMM with different concentrations of gallium showing that the mutant is more susceptible to gallium. *P < 0.05; ***P < 0.001.

We tested the combination of BPS, an iron chelator, and gallium in RPMI and AMM and calculated the FICI. The FICI mean was 0.33 and 0.28 for the combination in RPMI and AMM, respectively, indicating a strong synergism between gallium and the iron chelator (BPS) (Figures 5C,D). Our results indicate that iron-limiting conditions enhance the gallium antifungal activity. To investigate whether the fungistatic effect of gallium is related to iron metabolism, we used an *A. fumigatus* Δ*hapX* mutant. HapX is an essential transcription factor for iron assimilation under iron starvation conditions (Haas, 2014). We observed that the MIC of gallium for Δ*hapX* was slightly lower than for the wild-type strain (8.0 vs. 16.0 mg/L) in RPMI and AMM (Table 3), but there was no inhibition in iron-containing medium (GMM) (Table 3). As the MIC between the strains were not substantially different and could be due to impaired growth of the mutant in the media, we also calculated the normalized radial growth in AMM supplemented with gallium. The growth of Δ*hapX* was significantly lower than the wild-type strain at all concentrations of gallium tested (Figure 6). Together, these data show that the gallium anti-*Aspergillus* effect is more pronounced in an iron-deficient strain.

**Table 3:** MIC (mg/L) of gallium nitrate for *A. fumigatus* CEA10 wild-type and Δ*hapX*.

**Medium**	**Wt**	**Δ*hapX***
RPMI	16.0	8.0
AMM	16.0	8.0
GMM	>512.0	>512.0

We also tested the combination between gallium and systemic antifungals against Δ*hapX*. FICI showed that gallium interacts indifferently with voriconazole, posaconazole, and amphotericin B in Δ*hapX* as well (Table 3). The interaction with caspofungin, however, was synergistic and more evident in Δ*hapX* than in the wild-type strain (FICI means = 0.47 vs. 0.78, respectively) (Table 4).

**Table 4:** Fractional inhibitory concentration index (FICI) of gallium nitrate and azoles, caspofungin, and amphotericin B against *A. fumigatus* wild-type and Δ*hapX*.

**Drug**	**Strain**	**FICI at different gallium (mg/L)**							
		**0.125**	**0.25**	**0.5**	**1.0**	**2.0**	**4.0**	**8.0**	**Mean**
Voriconazole	Wt	1.0	0.51	0.53	0.56	0.62	0.75	0.75	0.67
	*ΔhapX*	1.0	1.0	1.0	1.1	1.2	0.75		1.0
Posaconazole	Wt	1.0	1.0	1.0	1.0	1.1	0.75	1.0	0.78
	*ΔhapX*	1.0	1.0	0.56	0.62	0.75	0.75		0.54
Caspofungin	Wt	1.0	1.0	1.0	1.0	1.0	0.51	0.04^*^	0.78
	*ΔhapX*	1.0	0.53	0.31^*^	0.18^*^	0.31^*^			0.47^*^
Amphotericin B	Wt	0.51	0.51	1.0	1.0	0.62	0.75	0.56	0.72
	*ΔhapX*	0.51	0.53	1.0	1.1	1.2	1.0		0.91

* *Synergistic interaction (in bold)*.

### Antifungal Effect of Gallium Against Other Fungal Pathogens

We investigated the inhibition of other important fungal pathogens. The MIC of gallium in RPMI for different species of drug-susceptible *Candida* (MIC for antifungal drugs in Table S2) ranged from 16.0 to 256.0 mg/L (MIC_50_ = 64.0 and MIC_90_ = 128.0 mg/ml), being more effective against *C. albicans, C. haemulonii, C. glabrata*, and *C. parapsilosis* (Table 5). The antifungal effect of gallium was also tested against multidrug resistant *Candida*. The lower MIC was obtained against multidrug-resistant *C. haemulonii* strains (range 16.0–32.0 mg/L) (Table 6). For *C. duobushaemulonii*, 128.0 mg/L of gallium was able to inhibit the fungal growth and for multidrug-resistant *C. glabrata* strains, the MIC range was 128–256 mg/L (Table 6). Regarding *C. auris*, the strains 473/2015, 490/2015, 501/2015, and 502/2015 were completely inhibited by gallium at concentration of 128–256 mg/L. However, the strains 467/2015, 470/2015, and 484/2015 survived the challenge at the higher concentration tested (Table 6). For all pathogens, gallium presented a fungistatic activity (Tables 5, 6). These results indicate that gallium has a potential antifungal activity against several important fungal pathogens.

**Table 5:** MIC (mg/L) of gallium nitrate in RPMI for *Candida* drug-susceptible strains.

**Strain**	**MIC (mg/L)**	**Fungicide or fungistatic effect**
*Candida albicans* ATCC 90025	16.0	Fungistatic
*C. albicans* 16	32.0	Fungistatic
*C. albicans* 83	64.0	Fungistatic
*C. albicans* 106	64.0	Fungistatic
*C. albicans* 123	64.0	Fungistatic
*C. glabrata* ATCC 90030	32.0	Fungistatic
*C. glabrata* 614	32.0	Fungistatic
*C. glabrata* 636	64.0	Fungistatic
*C. glabrata* 558	64.0	Fungistatic
*C. parapsilosis* ATCC 22019	32.0	Fungistatic
*C. krusei* ATCC 6558	256.0	Fungistatic
*C. auris* CBS 10913	128.0	Fungistatic
*C. haemulonii* sensu stricto 768	32.0	Fungistatic
*C. haemulonii* sensu stricto 3834	64.0	Fungistatic
*C. haemulonii* sensu stricto 6083	32.0	Fungistatic
*C. haemulonii* sensu stricto 145/18	64.0	Fungistatic
MIC_50_	64.0	–
MIC_90_	128.0	–

*MIC_50/90_, values that inhibited 50 and 90% of the strains, respectively*.

**Table 6:** MIC (mg/L) of gallium nitrate in RPMI for multidrug-resistant *Candida*.

**Strains**	**MIC (mg/L)**
*C. haemulonii* sensu stricto CBS 5149	16.0
*C. haemulonii* sensu stricto 585/2015	16.0
*C. haemulonii* sensu stricto 767/2015	16.0
*C. haemulonii* sensu stricto 9700B	32.0
*C. haemulonii* var *vulnera* 583/2015	16.0
*C. haemulonii* var *vulner* 9873	32.0
*C. duobushaemulonii* 546/2015	128.0
*C. duobushaemulonii* 6983	128.0
*C. auris* 470/2015	> 512.0
*C. auris* 484/2015	> 512.0
*C. auris* 467/2015	> 512.0
*C. auris* 473/2015	256.0
*C. auris* 490/2015	256.0
*C. auris* 502/2015	256.0
*C. auris* 501/2015	128.0
*C. glabrata* 8622 A	128.0
*C. glabrata* 8622 B	256.0
*C. glabrata* 8622 C	256.0
*C. glabrata* 8622 D	256.0
MIC_50_	128.0
MIC_90_	> 512.0

*MIC_50/90_, values that inhibited 50 and 90% of the strains, respectively*.

## Discussion

Fungal infections kill millions of people annually (Almeida et al., 2019). This high fatality rate can be explained by a combination of factors including host conditions, time to diagnose the infection, as well as the limited antifungal drugs available and the emergence of resistant strains (Colombo et al., 2017; Perfect, 2017). An unconventional approach to fight fungal infection is to exploit nutritional vulnerabilities of the fungal metabolism (Li et al., 2018), similar to what has been used in cancer and antibacterial therapies (Chitambar, 2010; Goss et al., 2018).

In this paper, we have tested the gallium nitrate III antifungal effect against azole-sensitive and -resistant *A. fumigatus* strains. Our results showed that gallium presents a fungistatic activity against *A. fumigatus* conidia and its inhibitory effect is drug-concentration-dependent as revealed by the time kill curve. Gallium anti-*Aspergillus* effect appears to be hampered by the presence of iron since it is unable to inhibit fungal growth in medium containing iron. This result is in agreement with other studies showing that gallium can inhibit bacterial growth only in iron-depleted medium (Chitambar, 2010; Goss et al., 2018). To verify the hypothesis that gallium can interfere in cell iron homeostasis in *A. fumigatus*, first we challenged the conidia with different concentrations of gallium at increasing levels of FeSO_3_ and we observed that iron presence negatively affected the antifungal activity. Then, we detected a strong synergism when an iron chelator (BPS) was combined with gallium, proving that gallium antifungal activity is iron-dependent.

The redox properties of iron are important in several cellular processes, such as electron transport chains, respiration, DNA synthesis, Krebs cycle, and oxygen transport/storage (Li et al., 2018). This importance shows why pathogens require adequate iron concentration inside the host to grow and express their virulence factors. On the other hand, hosts limit iron by compartmentalization or through iron complexation with proteins, such as hemoglobin, transferrin, and lactoferrin (Haas, 2014; Li et al., 2018). Furthermore, additional iron restriction mechanisms occur during infection in a process named “nutritional immunity” (Cassat and Skaar, 2013). The activity of these host defenses, together with the insolubility of iron in aerobic environments, explain the extremely low iron concentration found *in vivo* (~10^−20^ M) (Bullen et al., 2005).

To counterbalance the iron limitation inside the host, *A. fumigatus* acquired mechanisms for obtaining iron, which are controlled at the transcriptional level by two transcription factors, SreA (important for iron-replete conditions) and HapX (important for iron-depletion condition) (Haas, 2014). In our work, we used Δ*hapX* to investigate if a strain unable to grow in total absence of iron would be more susceptible to gallium. Our findings showed that this transcription factor is important to tolerate more gallium because the mutant presented lower MIC and radial growth than the wild-type strain.

Antifungal combination is a common approach aiming to (i) potentialize the antimicrobial effect; (ii) reduce the dose of single drug usage with increased drug efficacy, consequently decreasing the drug toxicity; and (iii) hinder the development of resistant strains (Spitzer et al., 2017; Simm and May, 2019). We tested the combination between gallium and antifungal agents against *A. fumigatus* and observed that gallium had a synergistic effect with caspofungin, a second-line therapy against invasive aspergillosis. In addition, gallium decreased the CPE. Other studies have also exploited and proved the synergistic effect between substances that affect iron homeostasis and azoles (Gautam et al., 2011; Simm and May, 2019), unlike gallium, which had the same effect only with caspofungin.

The most severe, common, and difficult-to-treat mycoses are those caused by three fungi: *A. fumigatus, Candida* spp., and *Cryptococcus* spp. (Brown et al., 2012a; Perfect, 2017). As gallium had antifungal activity against *A. fumigatus*, we tested it against *Candida*. Gallium inhibited the growth of all species tested, being more effective against *C. albicans, C. haemulonii, C. glabrata*, and *C. parapsilosis*. These differences may reflect distinct forms of how these species combat iron disruption inside the cell. Furthermore, gallium inhibited multidrug-resistant *C. haemulonii, C. duobushaemulonii, C. glabrata*, and *C. auris* strains, demonstrating that it may be a potential agent for treating infections caused by emergent pathogens.

We were able to demonstrate that gallium nitrate III exhibits *in vitro* antifungal inhibitory activity against human fungal pathogens, including azole-resistant *A. fumigatus* and multidrug-resistant *Candida* strains. More studies need to be done addressing whether the effect that we have reported also manifests *in vivo*. In addition, it could be interesting to investigate if gallium nitrate could act against bacteria and fungi in polymicrobial infection, as those that frequently happen in patients with cystic fibrosis (Zhao and Yu, 2018).

In this study, we have investigated the antifungal effect of gallium nitrate III, which is an FDA-approved drug (Ganite^TM^), and the first generation of gallium compounds. Additionally, there are several other gallium-based metallodrugs and novel gallium agents available and in development (Chitambar, 2010; Hijazi et al., 2018) that can exert distinct antimicrobial effects (Hijazi et al., 2018). In the future, studies should focus on testing if other gallium compounds are more effective and specific against fungal cells and the safety of those drugs for the host.

In conclusion, gallium has a fungistatic effect against mold and yeasts, like *A. fumigatus* and *Candida* spp. This effect seems to be due to iron disruption caused by gallium inside the cell. In addition, it has a synergistic interaction with caspofungin against *A. fumigatus*.

## Data Availability Statement

The datasets generated for this study are available on request to the corresponding author.

## Author Contributions

RB and GG conceived and designed the experiments. RB, LR, and CV performed the experiments. RB, LR, CV, and GG analyzed the data. GG, KL, and AC contributed reagents, materials, and analysis tools. RB, AC, and GG contributed to the writing of the manuscript.

### Conflict of Interest

The authors declare that the research was conducted in the absence of any commercial or financial relationships that could be construed as a potential conflict of interest.
