# Supplementary material for: Potential of Gallium as an Antifungal Agent
Source: Front Cell Infect Microbiol. 2019 Dec 11;9:414. doi: 10.3389/fcimb.2019.00414 (PMC6917619; doi:10.3389/fcimb.2019.00414)
Supplement: Supplementary file 1 [file Data_Sheet_1.pdf]

Table S1. Minimum inhibitory concentration (MIC- mg/mL) of azoles drugs for *Aspergillus fumigatus* strains.

Strain/parameter	Voriconazole	Posaconazole	Itraconazole
Aspergillus fumigatus CEA10[S]	0.50	1.00	0.50
A. fumigatus Af293 [S]	0.50	1.00	1.00
A. fumigatus AfS35 [S]	0.50	1.00	0.50
A. fumigatus CEA17 [S]	0.25	0.50	0.50
A. fumigatus CYP-15-75[AR]	4.00	1.00	>8.00
A. fumigatus CYP-15-91[AR]	4.00	1.00	>16.00
A. fumigatus CYP-15-93[AR]	4.00	1.00	>8.00
A. fumigatus CYP-15-106 [AR]	4.00	0.50	>8.00
A. fumigatus CYP-15-108[AR]	8.00	1.00	>16.00
A. fumigatus CYP-15-109[AR]	>8.00	1.00	2.00
A. fumigatus CYP-15-115[AR]	4.00	0.50	>16.00
A. fumigatus CYP-15-117[AR]	0.25	0.50	>16.00

S= susceptible, AR=Azole-resistant

Table S2. Minimum inhibitory concentration (MIC- mg/mL) of amphotericin B, fluconazole and caspofungin for susceptible *Candida* spp.

Strains	Amphotericin B	Fluconazole[a]	Caspofungin
Candida haemulonii sensu stricto 768	0.12	0.12	0.12
C. haemulonii sensu stricto 3834A	0.50	0.50	0.06
C. haemulonii sensu stricto 6083	0.50	0.25	0.06
C. haemulonii sensu stricto 145/18	0.50	0.12	0.03
C. albicans ATCC 90025	0.06	2.00	0.12
C. albicans 16	0.06	0.12	0.12
C. albicans 83	0.06	0.25	0.12
C. albicans 106	0.06	0.12	0.06
C. albicans 123	0.06	0.12	0.06
C. globrata ATCC 90030	0.25	0.25	0.06
C. glabrata 614	0.06	0.12	0.06
C. glabrata 636	0.06	0.50	0.12
C. glabrata 558	0.06	0.25	0.12
C. parapsilosis ATCC 22019	0.06	0.50	0.25
C. krusei ATCC 6558	0.06	4.00	0.25
C. auris CBS 10913	0.50	2.00	0.06

a- MIC endpoint considering 50% of growth inhibition
